# Benefits of Massive Open Online Course Participation: Deductive Thematic Analysis

**DOI:** 10.2196/17318

**Published:** 2020-07-08

**Authors:** Elizabeth R Blum, Terese Stenfors, Per J Palmgren

**Affiliations:** 1 Department of Learning, Informatics, Management and Ethics Karolinska Institutet Stockholm Sweden

**Keywords:** MOOC, MOOC evaluation, qualitative, thematic analysis, learner, online education, Kirkpatrick framework, outcomes, MOOC outcomes

## Abstract

**Background:**

Massive open online courses (MOOCs), as originally conceived, promised to provide educational access to anyone with an internet connection. However, the expansiveness of MOOC education has been found to be somewhat limited. Nonetheless, leading universities continue to offer MOOCs, including many in the health sciences, on a number of private platforms. Therefore, research on online education must include thorough understanding of the role of MOOCs. To date, studies on MOOC participants have focused mainly on learners’ assessment of the course. It is known that MOOCs are not reaching the universal audiences that were predicted, and much knowledge has been gained about learners’ perceptions of MOOCs. However, there is little scholarship on what learners themselves gain from participating in MOOCs.

**Objective:**

As MOOC development persists and expands, scholars and developers should be made aware of the role of MOOCs in education by examining what these courses do offer their participants. The objective of this qualitative synthesis of a set of MOOC evaluation studies was to explore outcomes for MOOC learners, that is, how the learners themselves benefit from participating in MOOCs.

**Methods:**

To explore MOOC learners’ outcomes, we conducted a qualitative synthesis in the form of a deductive thematic analysis, aggregating findings from 17 individual studies selected from an existing systematic review of MOOC evaluation methods. We structured our inquiry using the Kirkpatrick model, considering Kirkpatrick levels 2, 3, and 4 as potential themes in our analysis.

**Results:**

Our analysis identified six types of Kirkpatrick outcomes in 17 studies. Five of these outcomes (learning/general knowledge, skills, attitudes, confidence, and commitment) fit into Kirkpatrick Level 2, while Kirkpatrick Level 3 outcomes concerning behavior/application were seen in four studies. Two additional themes were identified outside of the Kirkpatrick framework: culture and identity outcomes and affective/emotional outcomes. Kirkpatrick Level 4 was not represented among the outcomes we examined.

**Conclusions:**

Our findings point to some gains from MOOCs. While we can expect MOOCs to persist, how learners benefit from the experience of participating in MOOCs remains unclear.

## Introduction

When the first massive open online course (MOOC) was offered in 2008, the MOOC format—free, online, and open to anyone with an internet connection—was touted as revolutionary for its potential to democratize access to educational opportunities due to its theoretically universal availability [1-3]. The earliest MOOCs used a connectivist paradigm in which the course was built from networks of online resources and relied on openness and participation from learners. These so-called cMOOCs had the potential to allow learners to participate in their own education outside the traditional, face-to-face classroom setting and to connect with learners worldwide [4]. Extended MOOCs (xMOOCs) brought the MOOC format back to a more traditional structure, with instructors determining the content while still providing “open” availability to anyone with internet access. In practice, there are limits to what this expansive availability has accomplished [2,5]. However, as MOOCs persist, it is useful to explore their role in education by examining what they do offer their participants.

Learning is a complex phenomenon that can be described from different perspectives. Understanding learning is about understanding not only learning processes but also the conditions that influence—and are influenced by—the learning process [6]. In this paper, learning is understood from a constructivist and social-constructivist perspective in which reality and new understanding are constructed by learners on the basis of their previous knowledge, perceptions, and experiences. Learning thus consists of contextual aspects (ie, teachers present information in a way that enables learners to construct meaning on the basis of their own experiences, with a focus on situating learning in an authentic activity); cognitive aspects (ie, recognizing individuals’ perception, memory, and meaning-making); and social aspects (ie, converging on learning as a social activity that occurs through interactions between the learner and others) [7,8]. This conception of learning thus reintegrates the artificial and no longer useful distinction between cMOOCs and xMOOCs [9].

A number of systematic reviews have examined MOOCs [4,10-17]. These reviews indicate that much research on MOOCs focuses on evaluating noncompletion rates and retention vs attrition; learner motivation and engagement as well as other behavioral elements, and how these relate to retention and achievement; implications of the latter for MOOC design; and learners’ own assessments of the courses [3,4,10,12,16,18]. Research also points to a lack of studies on learners’ own experiences and outcomes [3,4,10]; however, there are some exceptions [14,19]. For example, in their review, Pilli and Admiraal [19] investigated MOOC learner outcomes with the intention of informing MOOC course design. Joksimovic et al [14] argued that outcomes and learner engagement are commonly differentiated in the MOOC literature; however, their systematic review proposes an approach that reconnects the two, especially for MOOCs that do not include assessments (eg, cMOOCs as originally conceived). Joksimović et al [14] built on a model by Reschly and co-workers [20] that conceives of learning outcomes as “proximal” or “distal,” with academic, social, and affective outcomes within each; they modified this model for the “nonformal, digital educational settings” of MOOCs [14]. Despite their work on outcomes, Joksimovic and colleagues reiterated the finding that attempts to measure or evaluate the benefits to learners of participating in MOOCs have been mostly limited to date.

Another systematic review by Rowe et al [17] investigated the utility of open online courses (OOCs, including MOOCs) in health professions education. They evaluated the available research with a framework that included five “outcome” categories, including effectiveness (increase in learner knowledge), learner experiences, feasibility, pedagogy, and economics; they concluded that the available evidence neither unequivocally supports nor refutes the use of such courses. Their review was limited to the health professions; however, it highlighted the absence of rigorous research on MOOCs and the concurrent persistence of these courses. Their “effectiveness” category further highlighted the absence of research on benefits to MOOC learners, specifically in the health professions. They argued that the application of MOOCs in health professions education should be limited until a great deal more quality research is performed [17].

In their recent systematic review, Alturkistani et al [21] also added to the discourse on MOOC evaluation methods. Alturkistani et al identified three “evaluation-focused categories” among the studies they reviewed: learner-focused, teaching-focused, and MOOC-focused [21]. We approached this review as a jumping-off point to further synthesize understanding of MOOC learner outcomes. Here, we unpack the learner-focused category in [21] and, more specifically, the “learning outcomes and experience” subcategory to investigate the learner outcomes for the included MOOCs. In our study, “learner outcomes” are direct statements that describe the knowledge, skills, and attitudes that learners have demonstrated or are expected to reliably demonstrate when successfully completing a course. Learner outcomes is an understudied area that warrants further investigation, as MOOCs are a learning environment distinct from traditional classrooms and even other forms of e-learning, and they continue to be embraced as an educational modality [22].

Thus, despite their persistence, MOOCs have not lived up to the early expectation that they would allow widespread, nearly universal access to education. For example, there is consistent evidence that learners who use MOOCs, and indeed those who are more likely to complete them, are generally more educated and affluent [1,23,24]. There is also insufficient evidence that MOOCs are useful in areas such as health professions education [17]. MOOC learners are heterogeneous along numerous dimensions, including native language, prior training, age, economic status, and geographic location [24]. The heterogeneity of the expectations and goals of MOOC learners has also undoubtedly contributed to the difficulty of evaluating MOOCs and characterizing their benefits, a difficulty that is illustrated below in the heterogeneity of the studies reviewed. Thus, if MOOCs are not, in practice, democratizing education, and they have not lived up to traditional learning settings for at least some professional fields, what are they offering? In this study, we focus our attention on what learners do gain from participating in MOOCs, including but not limited to performance measures; that is, we explore how learners benefit from the experience of participating in MOOCs, including and beyond outcomes directly related to learning.

## Methods

We conducted a qualitative synthesis in the form of a deductive thematic analysis, aggregating findings from individual studies, to explore MOOC learners’ outcomes. The datasets used and analyzed during the current study are available from the first author on reasonable request. To structure our inquiry, we relied on a commonly used framework for evaluating learning with applications in multiple learning and training settings: the Kirkpatrick model [25]. This model frames training on four levels: (1) *reaction*, (2) *learning*, (3) *behavior*, and (4) *results*. A more recent version [26] updates and clarifies the model, proposing that *reaction* includes customer satisfaction, engagement, and relevance; *learning* includes knowledge, skills, attitude, confidence, and commitment; and *behavior* refers to how the learner applies the learning “on the job.” The more recent version of the behavior level adds “processes and systems that reinforce, encourage, and reward performance of critical behaviors on the job” [26], which can be seen as catalysts for applying what has been learned. These processes and systems, which include job aids, coaching, work review, and incentive systems, are referred to in [26] as “required drivers” or factors that increase the likelihood that people will retain and apply what they have learned in a given setting, referred to as “required drivers”. *Results* are the targeted outcomes of the training, such as whether the results of the training are seen within an organization; the more recent version adds “leading indicators” (short-term measures that can indicate whether the results are likely to occur) [26].

The studies in the current synthesis derive from Alturkistani et al’s systematic review of MOOC evaluation methods [21]. Their review included studies from 2008 to 2018 that focused primarily on MOOC evaluation and studies that reviewed or applied MOOC evaluation methods. Both quantitative and qualitative studies were included, after a careful assessment of their methodological quality, as well as grey literature. During the last few years, the contribution of qualitative evidence has been acknowledged within research [27]. This is in line with the epistemological stance of this review. The complete search strategy and further details of the source review [21] can be found in [18]. Alturkistani *et al* [21] identified 3275 records; after a review procedure, the final review included 33 studies.

**Figure 1:**
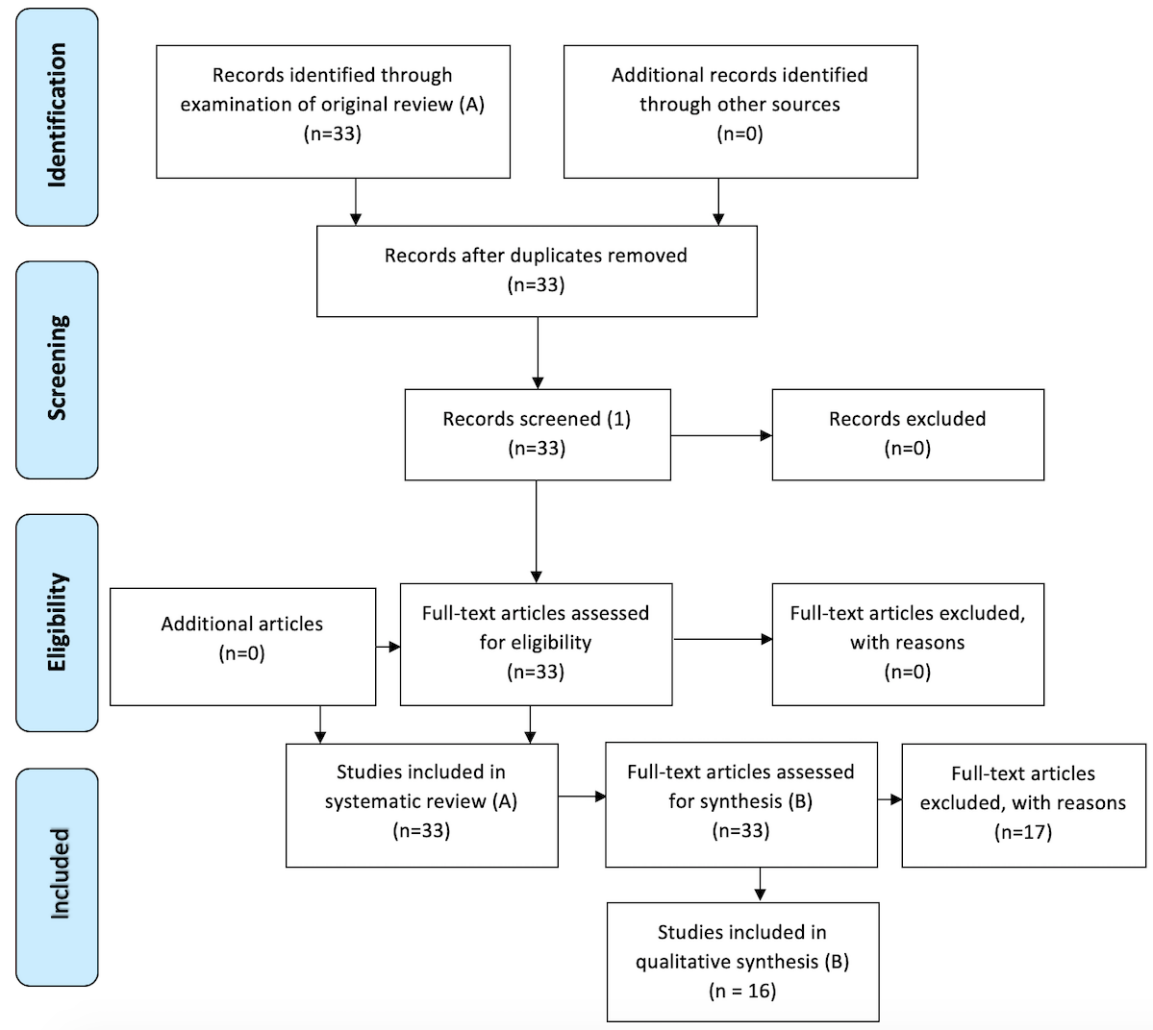
Preferred Reporting Items for Systematic Reviews and Meta-Analyses (PRISMA) flow diagram: systematic review (A) for a synthesis paper on MOOC learning outcomes (B). Modified from Alturkistani et al [21].

Specifically, Alturkistani et al’s “learning outcomes and experiences” subcategory was the basis for the current synthesis, as we looked at what learners gain from the experience of participating in MOOCs. This subcategory included 21 studies. We reviewed each paper in this category for findings that included learners’ outcomes*.* Each study was examined for outcomes specific to the learners themselves. We did not include measures of engagement, motivation, completion, or attrition in our analysis unless they were clearly tied to the outcomes for learners. In an additional step intended to capture all learner outcomes, we examined Multimedia Appendix 3 in Alturkistani et al’s review [21], which included all 33 studies. As a result of this review, we excluded 12 studies that did not include clear outcomes for learners (Figure 1), which left 21 studies for our analysis. As the analysis proceeded, we determined that the outcomes in 6 of these 21 studies were not clear enough to include. Notably, we did include one study [28] that was not included in Alturkistani et al’s “learner outcomes and experience” category. Of the resulting 16 studies for analysis, 4 had more than one outcome. Multimedia Appendix 1 describes this procedure in detail.

More specifically, in this qualitative synthesis, we performed a deductive thematic analysis [29] where the starting themes were the four Kirkpatrick levels. We extracted all outcomes from the 16 studies; we then placed these in Kirkpatrick level 2, 3, or 4. After this first coding, which was conducted by ERB, TS and PJP reviewed the results. Second, ERB further analyzed the findings in each category according to the subthemes within each Kirkpatrick level. Subsequently, the findings were discussed and subjected to adjustments until consensus among all investigators was reached. Although the aforementioned steps appear to be consecutively ordered, the process of analysis and search for patterns was in no way linear; rather, it was iterative and recursive. No software program was used to aid the analysis. The structure of our analysis allows for the possibility that the same study will have multiple outcomes and thus will appear under more than one level. Level 1 (*reaction*) in the Kirkpatrick model was not of interest to our investigation, as there is a great deal of existing research on learners’ assessment of MOOCs.

Outcomes that could not be matched with the Kirkpatrick levels were set aside for a separate inductive thematic analysis, which is presented as “Outcomes beyond Kirkpatrick.”

## Results

Our analysis resulted in six types of outcomes. These are summarized in Table 1 as framed by the levels in the Kirkpatrick model.

### Kirkpatrick Level 2: Learning

Our deductive analysis showed that 15/16 (94%) of the examined studies included one or more outcomes corresponding to Kirkpatrick Level 2. Thus, the Learning theme here incorporates concepts such as knowledge, skill, attitude, confidence, and commitment. Each subtheme is presented using the identified data and illustrated with supporting quotations.

#### Subtheme: Knowledge

Most of the Level 2 outcomes we identified were scores or survey items that assessed knowledge in some form. For example, in their MOOC on new media in teaching and learning, Chen et al [30] reported ”learning performance” via quiz scores and a final paper, for which the participants could earn ”Excellence Awards.” Four studies in our sample took a longitudinal view of learning outcomes via a pretest/posttest design. For example, Konstan et al [31] used a longitudinal design to test knowledge of technology that predicts preferences based on previous behavior (recommender systems technology); based on precourse and postcourse test scores within their MOOC, the gains in knowledge measured at the end of the course persisted at a 5-month follow-up in most cases. Further, in a MOOC designed to prepare medical students for global health experiences, Jacquet and colleagues [32] found an increase in post-MOOC compared to pre-MOOC test scores. Next, using average quiz and homework scores, Liang et al [33] reported an increase in quiz and homework scores enhanced by participation in online activities. Cross [34] used preassessments and postassessments to track changes in knowledge on a scale from “novice” to “expert,” while Colvin et al [35] reported improved scores on postcourse versus precourse tests in introductory physics, and Mackay et al [36] saw a postcourse increase in scores on their assessment of participants’ knowledge about animal welfare.

**Table 1:** Outcomes of MOOC studies framed by Kirkpatrick Level 2 or Level 3.

Kirkpatrick level, subtheme, and study			Data collection	Data analysis	Outcome variables	Outcome findings
**Level 2: Learning**						
	**1.** **General learning/change in knowledge**					
		Chen et al (2015) [30]	Scores on quizzes and final paper	Inferential statistics	Possible “Excellent Paper,” “Excellent Participation,” and “Excellent Group Member” awards	Learners received these awards if they fulfilled the criteria
		Konstan et al (2015) [31]	Three-part longitudinal design: precourse, postcourse, and 5-month follow-up “knowledge tests” and surveys	Inferential statistics; qualitative analysis	Assessed knowledge of recommender systems^a^	Gains in knowledge and 5-month retention of acquired knowledge
		Jacquet et al (2018) [32]	LMS^b^ data; pre-MOOC and post-MOOC knowledge tests	Inferential statistics	Score on knowledge test	Increased knowledge score from pretest to posttest
		Liang et al (2014) [33]	Assessments: quizzes and homework	Inferential statistics	Average assessment score	Increase in assessment score related to degree of participation
		Cross (2013) [34]	Precourse and postcourse surveys; LMS	Descriptive statistics	Knowledge: “novice” to “expert”^a^	Increase in knowledge
		Colvin et al (2014) [35]	Normalized gain between pretests and posttests in introductory physics; “ability” based on test items attempted, analyzed with Item Response Theory (IRT)	Inferential statistics	Comparison of pre-MOOC and post-MOOC physics knowledge and “ability”	Learning (measured via posttest score) across several cohorts identified using IRT
		MacKay et al (2016)[36]	Precourse and postcourse assessments of animal welfare knowledge	Inferential statistics	Scores on animal welfare knowledge assessment	Increased scores
	**2. Skill**					
		Brunton et al (2017)[37]	Weekly Likert scale quizzes during the MOOC: “individual digital readiness tools” and postcourse quiz	Descriptive statistics	Preparedness for online learning^a^	Self-assessed changes in preparedness for online learning
		Rubio (2015)[38]	Precourse and postcourse comprehensibility ratings	Inferential statistics	Spanish comprehensibility (language pronunciation)	Increased comprehensibility in postcourse ratings
		Stephens and Jones (2014) [39]	Precourse and postcourse surveys with mostly open-ended items	Content analysis	Skills discovery^a^	Technological skills
		Liu et al (2014) [40]	End-of-course surveys (Likert scale and open-ended); email interviews	Descriptive and thematic analysis (focused coding)	Three things students learned^a^	Skills in data visualization, critiquing, and creating infographics
	**3. Commitment**					
		Alturkistani et al (2018) [41]	Case studies; interviews	Thematic analysis	Learning achievement; use of information in the workplace^a^	Intention to apply knowledge
	**4. Attitude**					
		MacKay et al (2016) [36]	Multiple-choice quizzes; confidence and attitude surveys (mostly Likert scale)	Inferential statistics	Change in attitudes; certificate of achievement for completion^a^	Change in attitude
	**5. Confidence**					
		Hossain et al (2015) [28]	Ten-point scale; confidence-to-treat	Inferential statistics	Confidence to treat spinal cord injury^a^	Gains in confidence
		Cross (2013) [34]	Precourse/postcourse survey; LMS	Descriptive statistics	Confidence to apply learning^a^	Gains in confidence
		Mackness et al (2013) [42]	Interviews (face-to-face and email) and focus groups; assessment of microteaching	Qualitative case study approach	Confidence to participate in social learning environments^a^	Gains in confidence
		Lei et al (2015) [43]	Pre-MOOC and post-MOOC surveys; forum threads	Sentiment analysis	Identity and confidence^a^	Confidence in work; confidence to inspire
		Milligan and Littlejohn (2014) [44]	Interviews mid-MOOC	Qualitative analysis	Changes in practice^a^	Confidence about practices on the job
**Level 3: Behavior**						
	**Behavior/Application**					
		Milligan and Littlejohn (2014) [44]	Survey and interview	Qualitative analysis	Application of learning in professional practice^a^	Integrating new understanding in practice
		Lei et al (2015) [43]	pre-MOOC and post-MOOC surveys; forum threads	Sentiment analysis	Effects on learners and community^a^	Bringing knowledge back to community
		Cross (2013) [34]	Precourse/postcourse survey; LMS	Descriptive statistics	Changes in practice^a^	Implementation of tools in course design
		Konstan et al (2015) [31]	Follow-up interview and survey	Inferential statistics	Application of new recommender system skills^a^	Application of systems at work, school, business

^a^Includes a self-report.

^b^LMS: learning management system.

#### Subtheme: Skill

We found several examples of skill outcomes, including self-assessed preparedness (readiness for online education) [37] and improvement in Spanish language pronunciation and comprehensibility measured by pre-MOOC and post-MOOC assessments [38]. Further, participants in a library and information science MOOC were asked “What did you gain most from taking part in the MOOC?” Their responses included “Students gained new technological skills through their learning experience*”* [39]. Liu et al [40] found that learners gained skill through learning to “visualize data and critique infographics (and) learning visualization concepts and…tool use”; these were the most frequently cited “three things [students] had learned” in a journalism MOOC.

#### Subthemes: Commitment, Attitude, and Confidence

Other Level 2 outcomes were commitment, as shown through intention to apply knowledge [41]; attitude about animal welfare [36]; and confidence to treat patients, as measured in a randomized control trial study comparing a MOOC with a self-directed online learning module [28]. Additionally, Cross [34] reported that learners gained confidence with regard to applying what they had learned, and Mackness et al [42] also reported confidence to participate in various interactive learning activities:

They also gain the confidence to attend and contribute to live synchronous sessions, to openly share their work and ideas, and to cooperate and/or collaborate in social networking environments. “They shift from being consumers to producers.”

In their MOOC on Asian vernacular architecture, Lei et al [43] used a case study design to investigate learners’ postlearning experiences, asking, “How has the course influenced learners and their surrounding community?” This influence is reflected in the following learner’s experience:

It is through learning that I have gained the most confidence, in my identity and in my work. And I hope that this course would be the one of many stepping stones towards me being able to help inspire and nurture future generations….

Using a clinical trials MOOC, Milligan and Littlejohn [44] asked learners halfway through the course “to reflect on how their practice had changed as a result of the course.” Some learners had already seen an effect on their confidence and perspective: “These respondents reported a range of general benefits: that the course had given them a new perspective, had made them assured, or had helped them bring a greater criticality to their practice.” One participant stated, “I know why and why not…you have an overview, I cannot say I apply everything in my day to day work, but the fact that you feel more confident, for me, it helps a lot.” This outcome in turn intersects with Kirkpatrick Level 3, as discussed in the next section.

### Kirkpatrick Level 3: Behavior: Application

Our analysis found 4/21 studies (19%) with evidence of Level 3 outcomes. Level 3 includes application via critical behaviors plus the presence of outcomes that make it more likely that people will retain and apply what they have learned in a given setting (the abovementioned catalysts for application or “required drivers”).

In addition to effects on confidence (Level 2), Milligan and Littlejohn [44] found evidence of Level 3 outcomes from their clinical trials MOOC; in answer to the same question as above (how their practice changed as a result of the course), most learners reported having already incorporated their learning. For example, the respondent quoted above also reported immediate effects: “Well, it gives me a better understanding of why I do what I do…I understand why I have to submit my protocol or a complete or total submission to authorities, how a protocol has been developed.” [44] Another respondent said, “It is much, much better, I could address all of the challenges much better and make better decisions, and actually I participate with this CRO in developing the protocol and the study documents and everything.”

Lei and colleagues [43] described effects on how the learners brought their experience back to their communities, a behavioral application which reflects Kirkpatrick Level 3. For example, one participant from an area damaged by earthquakes reflected:

This course helped me to see the significance of the collapsed houses, temples, shrines, monuments and courtyards in a different angle which otherwise I would not have been able to see…I have already started contributing my knowledge with the local community as we come together to rebuild what has been destroyed.

Cross [28] described learners’ goals, including plans to implement tools from the MOOC in their course design; some learners reported having already done so, which is another example of application of the MOOC experience. Employing a longitudinal study design, Konstan et al [31] investigated MOOC learners’ application of course content (recommender systems technology). Kirkpatrick Level 3 behaviors are evident in the participants’ reports of incorporating the systems at work, school, or in entrepreneurial settings, and some also applied the underlying algorithms in other contexts.

### Kirkpatrick Level 4

In this qualitative synthesis, we did not find any data congruent with Kirkpatrick Level 4, which includes outcomes and “leading indicators.”

### Outcomes Beyond Kirkpatrick

Not all of the outcomes described in the studies are congruent or align well with the Kirkpatrick framework; hence, we present these outcomes separately here. After our inductive thematic analysis, we identified two themes among these outcomes: “culture and identity” outcomes and “affective/emotional” outcomes. Culture and identity outcomes included “insights about themselves through personal reflection about their learning styles, professional practices, and the ways they view the world” [39], as well as connection to a community, whether of fellow educators [39,42] or those with a shared cultural heritage [43]. Affective outcomes such as “excitement” and “inspiration” are evident in [39], where learners gained “inspiration, energy, and excitement about the field.”

## Discussion

### Principal Results

In this qualitative analysis, we explored the benefits that MOOCs in a broad range of subjects offer their participants. We synthesized the types of outcomes reported in a set of MOOC studies, including but not limited to outcomes that assess learning in some way. Using the Kirkpatrick model as a framework, the most prominent findings were that most of the MOOCs described in the included studies only had outcomes that could be categorized as Kirkpatrick Level 2. Kirkpatrick Level 3 outcomes were also represented, although these were not as common as Level 2 outcomes. We did not observe any Kirkpatrick Level 4 outcomes in the data we analyzed. If a MOOC were to aim for or result in Level 4 outcomes, we would expect to see changes at the organizational level. This might reveal itself in the form of implemented changes in policy in a health care setting after a group of managers participated in a policy MOOC, or in the case of higher education, a change in pedagogical training for educators after a MOOC was attended by several faculty members. Our complementary analysis of outcomes that did not align with Kirkpatrick yielded two additional themes.

### Previous Research

Previous research has shown that students generally perform better in face-to-face courses than in online courses [45], and several of the studies in our review used comparisons between MOOC and non-MOOC learning contexts. The studies analyzed in this study did not report outcomes that were unique to MOOCs; however, they did provide insight into what MOOCs do and do not offer to participants. For example, in a randomized control trial by Hossain et al [28] comparing a self-paced online course with an online course with MOOC-based guidance and study tips, improvement in knowledge of spinal cord injury treatment as well as gains in confidence to treat were observed after both courses; however, there was no advantage in the MOOC group. Additionally, Chen and coworkers [30] found no difference in scores on assignments between an online and an onsite version of a digital media course. Colvin et al [35] compared learning gains measured in their MOOC with learning gains in traditional settings; they found evidence of learning in the MOOC, in which scores were slightly higher than typical for a comparable lecture-based course but significantly lower than those seen in other courses with an “interactive engagement” component. In a finding that appears counter to the above, Rubio [38] found that improvement in language comprehensibility was greater in a MOOC compared to a face-to-face course. Finally, in their review, Rowe et al [17] looked specifically at the effectiveness of MOOCs in health professions education; they concluded that it cannot be said that MOOCs “enhance student learning” despite the proliferation of MOOCs and the “hype” about their potential. These contradictory findings suggest that when comparing MOOCs to other learning formats, the benefits of MOOCs remain unclear.

MOOCs were also expected to foster and build social networks. However, in reality, the amount of interaction among MOOC participants is often limited, and a small proportion of learners are usually responsible for most of this interaction. This finding was reinforced by the studies we examined [42,43,45]. However, there are social elements to MOOC participation, as discussed in the Outcomes Beyond Kirkpatrick section above. Joksimović and colleagues [14] proposed a model that may be a useful framework for illuminating some of the outcomes that do not readily fit with the Kirkpatrick framework. Their model considers social outcomes (along with academic and affective outcomes) in “immediate,” “course-level,” and “postcourse” settings. Since affective and social outcomes are evident in the studies critically analyzed here, it is worthwhile to consider them as benefits to MOOC participation, which may warrant additional research in its own right; the model proposed by Joksimović and colleagues [14] may be a useful starting point.

### Methodological Considerations

Using a well-known model to frame and lens our findings, in this study, we explored one understudied aspect of MOOCs that provides a view of what learners can gain from MOOCs. The richness of data using an in-depth secondary analysis of a small number of studies from a systematic review with broad subject matter, combined with frequent debriefing sessions and investigator triangulation, enhanced the credibility of the findings. We argue that qualitatively synthesizing existing data in an attempt to make sense of contextually and methodologically diverse findings is an important contribution to the scholarly literature. There are also some limitations to this study. Synthesizing both quantitative and qualitative data is a daunting task, as these data derive from very different paradigms. Thus, an important factor limiting the applicability of our findings is the problem with extracting results from eclectic and dissimilar studies, including qualitative and quantitative methods and grey literature, and attempting to contrast and compare them. The findings should thus be interpreted with due caution in light of this fact. Further, as our work builds on a previous review, we included only studies that were included therein. This may leave out some relevant studies, despite the rigorous inclusion criteria of the previous review. Finally, despite the frequent scholarly use of the Kirkpatrick framework, there are some inherent limitations to the model that also have implications for this work. It has been argued that the four-level model depicts an oversimplified view of learning and training effectiveness that does not take individual or contextual influences into account in the evaluation of the learning that occurs [46]. Thus, using the Kirkpatrick framework deductively as in this study and assorting “contextual” data into predefined themes was challenging. Further, Kirkpatrick’s model assumes that the four levels denote a causal chain in which positive reactions lead to greater learning and training, yielding greater transfer and, consequently, more positive results. While the Kirkpatrick model is vague about the causal relationships between level outcomes, it does imply that a simple causal relationship exists between the levels in the model [47]. Finally, in this study, we examined data that were not congruent with the framework but which are nonetheless important to the discussion of MOOC outcomes. For example, when considering the outcomes reported in the studies we reviewed, we chose not to include outcomes we viewed as belonging to Kirkpatrick Level 1, Reaction. This level is usually reserved for outcomes that reflect a participant’s reaction to a particular program or training. Since this may include how the participants “feel” about the program in question, Level 1 outcomes can certainly include an affective state in relation to the training. We found some outcomes that we described as “affective,” which included “feelings” such as excitement and inspiration. However, these feelings did not refer to the MOOC (training) itself. Instead, the “excitement” and “inspiration” were feelings about the subject of the MOOC as a result of the MOOC, which does not seem to us to fall clearly within Kirkpatrick Level 1. We believe that these feelings may even fall under Kirkpatrick Level 2 in the “Attitude” category; however, we made the conservative decision to separate them. Whether these feelings are part of a Kirkpatrick framework would be an interesting topic for further inquiry.

### Conclusions

Our findings point to some gains from MOOCs, and while we can expect MOOCs to persist, how learners benefit from the experience of participating in these courses remains unclear. This is especially true when comparing MOOCs to other learning modes, as evidenced by the comparative studies included in our sample. In our study, we looked for gains or benefits to MOOC learners in all subject areas, and we used the Kirkpatrick framework to explore what learners might gain. From a diverse set of studies, we found outcomes that included changes in knowledge, skills, attitude, and confidence as well as changes in behavior, increased excitement about a subject, and effects on cultural identity as a result of MOOC participation. Thus, beyond outcomes that can be classified as “learning,” such as increased knowledge or skill, it does appear that MOOCs provide some value for participants via the gains described above.

In contrast to systematic reviews of MOOC research, we carried out a deeper qualitative analysis of a set of studies from one systematic review that looked only at MOOC evaluation methods. Thus, as an extension of Alturkistani et al [21], we sought to identify MOOC outcomes that benefit the learner. With a qualitative investigation of a subset of studies on MOOC evaluation methods, we were able to apply the Kirkpatrick framework to identify a number of types of learner outcomes. However, as others have pointed out, the absence of systematic ways of measuring the benefits to learners is evident in our synthesis, and work remains to be done to determine the role of MOOCs and what they offer to participants and to the world.

## Appendix

Multimedia Appendix 1 Description of the analytic procedure for the qualitative synthesis.

## Abbreviations

cMOOC: connectivist massive open online course
LMS: learning management system
MOOC: massive open online course
OOC: open online course
PRISMA: Preferred Reporting Items for Systematic Reviews and Meta-Analyses
xMOOC: extended massive open online course
