# Supplementary material for: Benefits of Massive Open Online Course Participation: Deductive Thematic Analysis
Source: J Med Internet Res. 2020 Jul 8;22(7):e17318. doi: 10.2196/17318 (PMC7381083; doi:10.2196/17318)
Supplement: Multimedia Appendix 1 [file jmir_v22i7e17318_app1.docx]

**Appendix I**

**Description of analytic procedure for Blum et al. 2020 JMIR ms#17318**

In the present study, we:

1. Reviewed all **21^a^** studies in Table 1, “Learner outcomes and experience” category, in Alturkistani et al.’s Systematic Review [21].
2. Reviewed all remaining studies (those not included in Step 1, above) from the “Multimedia Appendix 3: Data abstraction form” in Alturkistani et al. [21]. This expanded the view to capture all possible learner outcomes reported in all studies included the systematic review. Total steps 1 and 2 = 33 studies^a^
3. Preliminarily categorized these 33 into Kirkpatrick levels 2, 3, 4 or “No outcomes”.
4. Removed 12 studies with no outcomes. Total remaining = 21 studies (does not overlap completely with 21 studies in Step 1.) See embedded Table.
5. Excluded six studies from these 21, based on our “outcomes” criteria. These six were included in Alturkistani et al.’s Table 1 “Learner outcomes and experience” (thus **21 – 6 = 15**):
   1. Milligan & Littlejohn (2017) [28]
   2. Lesjak & Florjancic (2014) [29]
   3. Li & Wan (2016) [42]
   4. Hudson et al. (2016) [46]
   5. Mee et al. (2016) [49]
   6. Liu et al. (2015) [38]
6. Added one (15 + 1 = 16): Hossain et al. 2015 [20] (Categorized under “Learner satisfaction” in Table 1 [21])^a^
7. Categorized the remaining 16 studies into K levels (Table 1 in the present paper)
8. Further divided the latter into subthemes where relevant.

^a^ All information in this description refers to Alturkistani et al. [21].

Table 1. Comparison of studies in included in ‘Learning outcomes

and experience’ [21] and studies in the present paper.

| Table 1 ‘Learning outcomes and experience’ [21]  ^b^ not included in present study | Studies reviewed in the present paper  ^c^ not included in Table 1, ‘Learning outcomes and experience’ [21] |
| --- | --- |
| Alturkistani et al. 2018 | Alturkistani et al. 2018 |
| Brunton et al. 2017 | Brunton et al. 2017 |
| Chen et al. 2015 | Chen et al. 2015 |
| Colvin et al. 2014 | Colvin et al. 2014 |
| Cross 2013 | Cross 2013 |
| Hudson et al. 2016^b^ | Hossain et al. 2015^c^ |
| Jacquet et al. 2018 | Jacquet et al. 2018 |
| Konstan et al. 2015 | Konstan et al. 2015 |
| Lei et al. 2015 | Lei et al. 2015 |
| Lesjak & Florjancic 2014^b^ | Liang et al. 2014 |
| Li & Wan 2016^b^ | Liu et al. 2014 |
| Liang et al. 2014 | MacKay et al. 2016 |
| Liu et al. 2014 | Mackness et al. 2013 |
| Liu et al. 2015^b^ | Milligan & Littlejohn 2014 |
| MacKay et al. 2016 | Rubio 2015 |
| Mackness et al. 2013 | Stephens & Jones 2014 |
| Mee et al. 2016^b^ |  |
| Milligan & Littlejohn 2014 |  |
| Milligan & Littlejohn 2017^b^ |  |
| Rubio 2015 |  |
| Stephens & Jones 2014 |  |
